# Supplementary material for: Altered microRNA Transcriptome in Cultured Human Liver Cells upon Infection with Ebola Virus
Source: Int J Mol Sci. 2021 Apr 6;22(7):3792. doi: 10.3390/ijms22073792 (PMC8038836; doi:10.3390/ijms22073792)
Supplement: Supplementary file 1 [file ijms-22-03792-s001.zip › Supplementary_File/C_ GO_Analysis_Results/16-30nt_go_Mayinga-96h-Huh7_vs_Control-96h-Huh7_down.mature_mirna_targets/MF_result(Human).html]

| GO.ID | Term | Ontology | Count | Pop.Hits | List.Total | Pop.Total | Fold.Enrichment | Pvalue | FDR | Enrichment.Score | Gene.Ratio | GENES |
| --- | --- | --- | --- | --- | --- | --- | --- | --- | --- | --- | --- | --- |
| GO:0022839 | ion gated channel activity | Molecular function | 17 | 335 | 382 | 17548 | 2.33114011096351 | 0.00113147417677167 | 0.583531062624863 | 2.94635535334296 | 0.0445026178010471 | P2RX2//GRIA3//GRIN2B//ANO8//ANO3//BEST1//GABRR1//HTR3E//KCNIP3//SCN3B//SCN1A//CACNG1//LRRC38//KCNA6//KCNQ4//KCNIP2//ABCC8// |
| GO:0022836 | gated channel activity | Molecular function | 17 | 338 | 382 | 17548 | 2.31044951826265 | 0.00124600027867852 | 0.583531062624863 | 2.9044818605432 | 0.0445026178010471 | P2RX2//GRIA3//GRIN2B//ANO8//ANO3//BEST1//GABRR1//HTR3E//KCNIP3//SCN3B//SCN1A//CACNG1//LRRC38//KCNA6//KCNQ4//KCNIP2//ABCC8// |
| GO:0005048 | signal sequence binding | Molecular function | 5 | 47 | 382 | 17548 | 4.88693327392225 | 0.00344837307674033 | 0.583531062624863 | 2.4623857543309 | 0.0130890052356021 | PEX5//KDELR1//NUP214//TOMM40L//KCNIP2// |
| GO:0034483 | heparan sulfate sulfotransferase activity | Molecular function | 3 | 15 | 382 | 17548 | 9.18743455497382 | 0.00383313068092386 | 0.583531062624863 | 2.41644637431813 | 0.00785340314136126 | NDST2//NDST3//HS6ST1// |
| GO:0015459 | potassium channel regulator activity | Molecular function | 5 | 49 | 382 | 17548 | 4.68746660968052 | 0.00413791691848329 | 0.583531062624863 | 2.38321823339152 | 0.0130890052356021 | LRRC38//SGK2//ARPP19//KCNIP3//KCNIP2// |
| GO:0017147 | Wnt-protein binding | Molecular function | 4 | 31 | 382 | 17548 | 5.92737713224118 | 0.00437009884121154 | 0.583531062624863 | 2.35950874021326 | 0.0104712041884817 | FZD2//TRABD2B//SFRP1//FZD3// |
| GO:0034185 | apolipoprotein binding | Molecular function | 3 | 16 | 382 | 17548 | 8.61321989528796 | 0.00464225363554124 | 0.583531062624863 | 2.33327113499024 | 0.00785340314136126 | VLDLR//LRP8//CANX// |
| GO:0045296 | cadherin binding | Molecular function | 15 | 324 | 382 | 17548 | 2.1267209617995 | 0.00511094139402215 | 0.583531062624863 | 2.29149909897016 | 0.0392670157068063 | YKT6//PTPRT//EIF4G2//MAPRE1//LARP1//ARFIP2//SERBP1//DBNL//LRRC59//PARVA//RTN4//SPTBN2//TLN1//YWHAB//SEPT7// |
| GO:0050839 | cell adhesion molecule binding | Molecular function | 20 | 489 | 382 | 17548 | 1.87882097238728 | 0.00541527749467701 | 0.583531062624863 | 2.2663792839474 | 0.0523560209424084 | ITGA5//THBS1//TLN1//JAM3//FERMT3//YKT6//PTPRT//EIF4G2//MAPRE1//LARP1//ARFIP2//SERBP1//DBNL//LRRC59//PARVA//RTN4//SPTBN2//YWHAB//SEPT7//NLGN1// |
| GO:0005216 | ion channel activity | Molecular function | 18 | 424 | 382 | 17548 | 1.95016299515954 | 0.00560051247706069 | 0.583531062624863 | 2.2517722308909 | 0.0471204188481675 | P2RX2//GRIA3//GRIN2B//ANO8//ANO3//BEST1//GABRR1//HTR3E//CACNG1//SCN3B//SCN1A//LRRC38//KCNA6//KCNQ4//KCNIP2//SLC26A1//KCNIP3//ABCC8// |
| GO:0072341 | modified amino acid binding | Molecular function | 6 | 75 | 382 | 17548 | 3.67497382198953 | 0.00583031570836107 | 0.583531062624863 | 2.23430792779899 | 0.0157068062827225 | SYT9//MARK1//THBS1//TLN1//MGST1//CPS1// |
| GO:0005515 | protein binding | Molecular function | 275 | 11565 | 382 | 17548 | 1.09232360683865 | 0.0059577054925065 | 0.583531062624863 | 2.22492096908683 | 0.719895287958115 | YKT6//CPLX1//CPLX3//GOSR2//ELK1//BHLHE40//CDC5L//PASD1//GNAO1//GNAZ//GNA14//NUCB1//THBS1//KIT//MAEA//KLHL2//DBNL//PARVA//SPTBN2//MLPH//MICAL2//VAV3//PARD6B//TAGAP//RALGPS2//ARHGEF6//FGFR2//GRIN2B//IL5RA//NCAM1//BCR//ARPP19//RND1//GRP//IGFBP1//TNFSF14//NDP//SFRP1//EDN1//BMP10//IL26//TSLP//ITGA5//RASL11B//TLN1//JAM3//FERMT3//CAMKK2//CTH//EWSR1//PPP3CB//RIT2//KCNQ4//IGFBP7//CCL16//CXCL14//PTPRT//GAS2//KIF2A//DAPK3//MAPRE1//PRKAA1//XRCC4//YWHAB//FGF11//HEYL//NAB1//NFKB1//INSIG2//PURA//PAXBP1//LARP1//PSMC6//CCDC6//SH3BP5//FZD2//TRABD2B//FZD3//RALBP1//LRP8//UBE4B//KMT2E//PEX5//SCNM1//TNKS2//TSPAN14//TTC28//UBQLN1//RHOF//MAPK4//AKAP7//BAG5//CARHSP1//PPP1R26//PPP1R3F//ARFIP2//SYT9//ABCC8//NLGN1//SEMA4G//METTL21A//DNAJB12//PPP4R2//TOMM34//RTN4//PALM//SEPT7//GABPA//VLDLR//CANX//FOXP3//PIK3IP1//ASF1B//RAG1//ANP32E//ALAD//P2RX2//SULT4A1//MCIDAS//CREBZF//SNRPA//TRIM21//CNTN2//BEST1//DDX39B//PIP4K2C//RNF135//FBP2//OXSR1//MYOM3//EXD1//MGST1//ASCL1//TTK//CREB3L3//NDN//UBXN2B//TP53INP2//PFDN1//LRRC38//KCNIP3//KCNIP2//SCN3B//EIF4G2//SERBP1//LRRC59//EFNA3//TAF8//H3F3B//ANO3//BRK1//NDFIP2//NDFIP1//ARPC3//ACTR2//AKAP10//PTAFR//IARS//DPYS//IFRD2//CDK2AP1//TOMM40L//DLX5//PRDM4//PRDM12//CD81//TIMM23//PYURF//EMC8//PPIH//TGOLN2//TMED2//SNRNP27//NUDT4//POLI//VPS45//AP1S1//SLAIN1//MSI2//UBXN10//LYPD6//SYNPR//PACRGL//LSM11//CPS1//SRXN1//BROX//USP54//PATE1//EIF4A2//TET3//FBXO45//ERH//SKA3//FOXG1//FAM168A//MGRN1//SATB2//ATP1B4//HAAO//ZNF281//LDOC1//PAN3//FAM32A//NAT9//MYCBP//PTPN18//GPR3//ANKRD52//LIN9//C16ORF72//WDR91//ST8SIA5//CD209//EHD3//CDR2L//HMGB3//HOXC9//IL4R//FAM111B//REP15//C16ORF87//LGALS3BP//MAS1//MEA1//SMCO3//ASPH//MTAP//NFYA//PAX5//UFM1//C9ORF78//PHYH//POLR2E//SGTB//FAM206A//TMEM248//VPS53//THUMPD1//GPATCH2L//MAP7D1//GRIPAP1//CYSLTR2//ANKRD50//LYRM2//PTBP1//GATAD2B//TBC1D24//WDFY1//DDX55//PCTP//RAB2A//CEP85//USP46//SMPD1//TMPRSS2//C1QA//UBE2D3//UBE2E1//VSNL1//PAX8//CACNG1//TNFAIP8L2//AKIRIN1//NUP214//CASP7//OGT//CGNL1//CYP4F2//KRT38//SESTD1//ZW10//NREP//EEF1E1//VPS26A// |
| GO:0015267 | channel activity | Molecular function | 19 | 463 | 382 | 17548 | 1.88511076181968 | 0.00639939201381619 | 0.583531062624863 | 2.19386128501414 | 0.049738219895288 | P2RX2//GRIA3//GRIN2B//ANO8//ANO3//BEST1//GABRR1//HTR3E//KCNIP3//SCN3B//SCN1A//CACNG1//LRRC38//KCNA6//KCNQ4//KCNIP2//SLC26A1//ABCC8//TOMM40L// |
| GO:0022803 | passive transmembrane transporter activity | Molecular function | 19 | 464 | 382 | 17548 | 1.88104802310886 | 0.00654307721158964 | 0.583531062624863 | 2.1842179547789 | 0.049738219895288 | P2RX2//GRIA3//GRIN2B//ANO8//ANO3//BEST1//GABRR1//HTR3E//KCNIP3//SCN3B//SCN1A//CACNG1//LRRC38//KCNA6//KCNQ4//KCNIP2//SLC26A1//ABCC8//TOMM40L// |
| GO:0022838 | substrate-specific channel activity | Molecular function | 18 | 437 | 382 | 17548 | 1.8921489930152 | 0.00756219947577129 | 0.583531062624863 | 2.12135187099832 | 0.0471204188481675 | P2RX2//GRIA3//GRIN2B//ANO8//ANO3//BEST1//GABRR1//HTR3E//CACNG1//SCN3B//SCN1A//LRRC38//KCNA6//KCNQ4//KCNIP2//SLC26A1//KCNIP3//ABCC8// |
| GO:0005229 | intracellular calcium activated chloride channel activity | Molecular function | 3 | 19 | 382 | 17548 | 7.25323780655828 | 0.00765434264245576 | 0.583531062624863 | 2.11609210072178 | 0.00785340314136126 | ANO8//ANO3//BEST1// |
| GO:0061778 | intracellular chloride channel activity | Molecular function | 3 | 19 | 382 | 17548 | 7.25323780655828 | 0.00765434264245576 | 0.583531062624863 | 2.11609210072178 | 0.00785340314136126 | ANO8//ANO3//BEST1// |
| GO:0031683 | G-protein beta/gamma-subunit complex binding | Molecular function | 3 | 20 | 382 | 17548 | 6.89057591623037 | 0.00886174647924935 | 0.638045746505953 | 2.05248067861472 | 0.00785340314136126 | GNAO1//GNAZ//GNA14// |
| GO:0001614 | purinergic nucleotide receptor activity | Molecular function | 3 | 21 | 382 | 17548 | 6.56245325355273 | 0.0101742983579687 | 0.657179827195086 | 1.99249553097442 | 0.00785340314136126 | GPR34//PTAFR//P2RX2// |
| GO:0016502 | nucleotide receptor activity | Molecular function | 3 | 21 | 382 | 17548 | 6.56245325355273 | 0.0101742983579687 | 0.657179827195086 | 1.99249553097442 | 0.00785340314136126 | P2RX2//GPR34//PTAFR// |
| GO:0042813 | Wnt-activated receptor activity | Molecular function | 3 | 22 | 382 | 17548 | 6.26415992384579 | 0.0115936436446147 | 0.657179827195086 | 1.93578005272805 | 0.00785340314136126 | FZD2//SFRP1//FZD3// |
| GO:0005244 | voltage-gated ion channel activity | Molecular function | 10 | 198 | 382 | 17548 | 2.320059231054 | 0.0116629135999128 | 0.657179827195086 | 1.93319294166132 | 0.0261780104712042 | GRIN2B//CACNG1//LRRC38//KCNA6//KCNQ4//KCNIP2//ABCC8//KCNIP3//SCN3B//SCN1A// |
| GO:0022832 | voltage-gated channel activity | Molecular function | 10 | 198 | 382 | 17548 | 2.320059231054 | 0.0116629135999128 | 0.657179827195086 | 1.93319294166132 | 0.0261780104712042 | GRIN2B//KCNIP3//SCN3B//SCN1A//CACNG1//LRRC38//KCNA6//KCNQ4//KCNIP2//ABCC8// |
| GO:0001786 | phosphatidylserine binding | Molecular function | 4 | 42 | 382 | 17548 | 4.37496883570182 | 0.012897497759786 | 0.680200973883853 | 1.88949453888886 | 0.0104712041884817 | SYT9//MARK1//THBS1//TLN1// |
| GO:0008187 | poly-pyrimidine tract binding | Molecular function | 3 | 23 | 382 | 17548 | 5.99180514454814 | 0.0131211607616484 | 0.680200973883853 | 1.88202774346036 | 0.00785340314136126 | MSI2//RBMS3//PTBP1// |
| GO:0016709 | oxidoreductase activity, acting on paired donors, with incorporation or reduction of molecular oxygen, NAD(P)H as one donor, and incorporation of one atom of oxygen | Molecular function | 4 | 44 | 382 | 17548 | 4.17610661589719 | 0.0151228450727535 | 0.753815662088021 | 1.82036649698541 | 0.0104712041884817 | CYP7A1//CYP4F3//CYP4F2//MICAL2// |
| GO:0035586 | purinergic receptor activity | Molecular function | 3 | 25 | 382 | 17548 | 5.51246073298429 | 0.0165049723619605 | 0.764919994589724 | 1.78238519857136 | 0.00785340314136126 | P2RX2//GPR34//PTAFR// |
| GO:0042277 | peptide binding | Molecular function | 12 | 273 | 382 | 17548 | 2.01921638570853 | 0.0168528767341811 | 0.764919994589724 | 1.77332595573362 | 0.031413612565445 | PEX5//NLGN1//GRIA3//KDELR1//NUP214//PPIH//MAS1//TOMM40L//CD209//HLA-DPA1//MGST1//KCNIP2// |
| GO:0016595 | glutamate binding | Molecular function | 2 | 10 | 382 | 17548 | 9.18743455497382 | 0.0189479290076712 | 0.764919994589724 | 1.72243825111499 | 0.00523560209424084 | CPS1//GRIN2B// |
| GO:0000982 | transcription factor activity, RNA polymerase II proximal promoter sequence-specific DNA binding | Molecular function | 16 | 415 | 382 | 17548 | 1.77107172144074 | 0.0200731461909192 | 0.764919994589724 | 1.69738455246172 | 0.0418848167539267 | PRDM4//DLX5//ELK1//SATB2//GABPA//HEYL//NDN//PAX5//PLAG1//PAX8//ZNF281//PROX2//KCNIP3//ASCL1//BHLHE40//PAXBP1// |
| GO:0019706 | protein-cysteine S-palmitoyltransferase activity | Molecular function | 3 | 27 | 382 | 17548 | 5.10413030831879 | 0.0203319367390576 | 0.764919994589724 | 1.69182125023438 | 0.00785340314136126 | YKT6//ZDHHC20//GOLGA7B// |
| GO:0019707 | protein-cysteine S-acyltransferase activity | Molecular function | 3 | 27 | 382 | 17548 | 5.10413030831879 | 0.0203319367390576 | 0.764919994589724 | 1.69182125023438 | 0.00785340314136126 | YKT6//ZDHHC20//GOLGA7B// |
| GO:0071813 | lipoprotein particle binding | Molecular function | 3 | 27 | 382 | 17548 | 5.10413030831879 | 0.0203319367390576 | 0.764919994589724 | 1.69182125023438 | 0.00785340314136126 | LRP8//THBS1//VLDLR// |
| GO:0071814 | protein-lipid complex binding | Molecular function | 3 | 27 | 382 | 17548 | 5.10413030831879 | 0.0203319367390576 | 0.764919994589724 | 1.69182125023438 | 0.00785340314136126 | LRP8//THBS1//VLDLR// |
| GO:0015318 | inorganic molecular entity transmembrane transporter activity | Molecular function | 28 | 856 | 382 | 17548 | 1.50261780104712 | 0.0211910700916132 | 0.764919994589724 | 1.67384711204104 | 0.0732984293193717 | P2RX2//GRIA3//GRIN2B//ANO8//ANO3//BEST1//GABRR1//HTR3E//CACNG1//SCN3B//SCN1A//LRRC38//KCNA6//KCNQ4//KCNIP2//SLC26A1//KCNIP3//ABCC8//SLC1A6//SLC5A3//SLC39A2//SLC30A6//ATP1B4//ATP6V1G2//CTNS//SLC23A2//SLC27A4//SLC9B1// |
| GO:0005261 | cation channel activity | Molecular function | 13 | 316 | 382 | 17548 | 1.88982039896613 | 0.0215335646188476 | 0.764919994589724 | 1.66688407207366 | 0.0340314136125654 | P2RX2//GRIA3//GRIN2B//CACNG1//SCN3B//SCN1A//LRRC38//KCNA6//KCNQ4//KCNIP2//KCNIP3//ABCC8//HTR3E// |
| GO:0043522 | leucine zipper domain binding | Molecular function | 2 | 11 | 382 | 17548 | 8.35221323179439 | 0.02282904795928 | 0.764919994589724 | 1.64151219953265 | 0.00523560209424084 | DAPK3//CDC5L// |
| GO:0046873 | metal ion transmembrane transporter activity | Molecular function | 17 | 457 | 382 | 17548 | 1.70882261963408 | 0.0229577253889173 | 0.764919994589724 | 1.63907114327656 | 0.0445026178010471 | CACNG1//SCN3B//SCN1A//LRRC38//KCNA6//KCNQ4//KCNIP2//KCNIP3//ABCC8//SLC1A6//SLC5A3//SLC39A2//SLC30A6//ATP1B4//SLC9B1//GRIN2B//SLC23A2// |
| GO:0022890 | inorganic cation transmembrane transporter activity | Molecular function | 21 | 601 | 382 | 17548 | 1.60512583739143 | 0.0230184257631167 | 0.764919994589724 | 1.63792438121751 | 0.0549738219895288 | P2RX2//GRIA3//GRIN2B//CACNG1//SCN3B//SCN1A//LRRC38//KCNA6//KCNQ4//KCNIP2//KCNIP3//ABCC8//SLC1A6//SLC5A3//SLC39A2//SLC30A6//ATP1B4//ATP6V1G2//SLC9B1//HTR3E//SLC23A2// |
| GO:0005230 | extracellular ligand-gated ion channel activity | Molecular function | 5 | 75 | 382 | 17548 | 3.06247818499127 | 0.0237482679321563 | 0.769443881001864 | 1.62436805993123 | 0.0130890052356021 | P2RX2//GRIA3//GRIN2B//HTR3E//GABRR1// |
| GO:0008146 | sulfotransferase activity | Molecular function | 4 | 52 | 382 | 17548 | 3.53362867498993 | 0.0263627593167003 | 0.78092560940837 | 1.57900913530336 | 0.0104712041884817 | SULT4A1//NDST2//NDST3//HS6ST1// |
| GO:0005215 | transporter activity | Molecular function | 38 | 1263 | 382 | 17548 | 1.382116045483 | 0.0266091330010295 | 0.78092560940837 | 1.57496927573057 | 0.0994764397905759 | P2RX2//GRIA3//GRIN2B//ANO8//ANO3//BEST1//GABRR1//HTR3E//KCNIP3//SCN3B//SCN1A//CACNG1//LRRC38//KCNA6//KCNQ4//KCNIP2//SLC26A1//ABCC8//SLC1A6//CPLX1//CPLX3//SLC5A3//SLC39A2//SLC30A6//ATP1B4//SLC35A2//TOMM40L//PCTP//ATP6V1G2//VPS26A//SLC9B1//CTNS//SLC23A2//RALBP1//SLC27A4//TIMM23//VLDLR//STEAP2// |
| GO:0030548 | acetylcholine receptor regulator activity | Molecular function | 2 | 12 | 382 | 17548 | 7.65619546247819 | 0.0270057710953939 | 0.78092560940837 | 1.56854341780165 | 0.00523560209424084 | LYPD6//PATE1// |
| GO:0099602 | neurotransmitter receptor regulator activity | Molecular function | 2 | 12 | 382 | 17548 | 7.65619546247819 | 0.0270057710953939 | 0.78092560940837 | 1.56854341780165 | 0.00523560209424084 | PATE1//LYPD6// |
| GO:0016417 | S-acyltransferase activity | Molecular function | 3 | 31 | 382 | 17548 | 4.44553284918088 | 0.029323330679309 | 0.78092560940837 | 1.53278670205642 | 0.00785340314136126 | YKT6//ZDHHC20//GOLGA7B// |
| GO:0005200 | structural constituent of cytoskeleton | Molecular function | 6 | 107 | 382 | 17548 | 2.57591623036649 | 0.0295113955675505 | 0.78092560940837 | 1.53001025261609 | 0.0157068062827225 | ARPC3//ACTR2//KRT9//SPTBN2//TLN1//CCDC6// |
| GO:0000978 | RNA polymerase II proximal promoter sequence-specific DNA binding | Molecular function | 16 | 436 | 382 | 17548 | 1.68576780825208 | 0.0299000661476301 | 0.78092560940837 | 1.52432785088898 | 0.0418848167539267 | CREB3L3//ASCL1//BHLHE40//PRDM4//DLX5//ELK1//SATB2//ZNF281//GABPA//KCNIP3//NDN//PAX5//PLAG1//GATAD2B//PAX8//PAXBP1// |
| GO:0003924 | GTPase activity | Molecular function | 12 | 297 | 382 | 17548 | 1.8560473848432 | 0.0299591881572915 | 0.78092560940837 | 1.52346995945006 | 0.031413612565445 | GBP4//RND1//GNAO1//GNAZ//RABL3//RHOF//RAB2A//RIT2//RASL11B//RERGL//GNA14//SEPT7// |
| GO:0050321 | tau-protein kinase activity | Molecular function | 2 | 13 | 382 | 17548 | 7.06725734997986 | 0.0314634599301962 | 0.78092560940837 | 1.50219352119241 | 0.00523560209424084 | MARK1//PRKAA1// |
| GO:0000977 | RNA polymerase II regulatory region sequence-specific DNA binding | Molecular function | 22 | 662 | 382 | 17548 | 1.52661299251831 | 0.0330303244804197 | 0.78092560940837 | 1.48108715990339 | 0.0575916230366492 | PRDM4//DLX5//ELK1//SATB2//ZNF281//GABPA//KCNIP3//ASCL1//NDN//PAX5//PLAG1//GATAD2B//PAX8//PAXBP1//HEYL//H3F3B//NFKB1//NFYA//CREB3L3//BHLHE40//PURA//CDC5L// |
| GO:0005525 | GTP binding | Molecular function | 14 | 371 | 382 | 17548 | 1.73347821791959 | 0.0332475777263384 | 0.78092560940837 | 1.47823999001216 | 0.0366492146596859 | GBP4//ARFIP2//RND1//GNAO1//GNAZ//RABL3//EHD3//RHOF//RAB2A//RIT2//RASL11B//RERGL//GNA14//SEPT7// |
| GO:0005254 | chloride channel activity | Molecular function | 5 | 82 | 382 | 17548 | 2.80104712041885 | 0.0332527085951492 | 0.78092560940837 | 1.47817297351826 | 0.0130890052356021 | ANO8//ANO3//BEST1//SLC26A1//GABRR1// |
| GO:0001012 | RNA polymerase II regulatory region DNA binding | Molecular function | 22 | 665 | 382 | 17548 | 1.51972601661221 | 0.034461017520906 | 0.78092560940837 | 1.46267190340596 | 0.0575916230366492 | ASCL1//NFKB1//GATAD2B//PURA//CREB3L3//CDC5L//PRDM4//DLX5//ELK1//SATB2//ZNF281//GABPA//KCNIP3//NDN//PAX5//PLAG1//PAX8//PAXBP1//HEYL//H3F3B//NFYA//BHLHE40// |
| GO:0032550 | purine ribonucleoside binding | Molecular function | 14 | 374 | 382 | 17548 | 1.71957331242826 | 0.0352127438717283 | 0.78092560940837 | 1.45330013221692 | 0.0366492146596859 | GBP4//ARFIP2//RND1//GNAO1//GNAZ//RABL3//EHD3//RHOF//RAB2A//RIT2//RASL11B//RERGL//GNA14//SEPT7// |
| GO:0017048 | Rho GTPase binding | Molecular function | 8 | 173 | 382 | 17548 | 2.12426232484944 | 0.0358910226075239 | 0.78092560940837 | 1.44501416756596 | 0.0209424083769634 | VAV3//BCR//ARHGEF6//RALBP1//ARFIP2//BRK1//DAPK3//PARD6B// |
| GO:0001608 | G-protein coupled nucleotide receptor activity | Molecular function | 2 | 14 | 382 | 17548 | 6.56245325355273 | 0.0361879703165352 | 0.78092560940837 | 1.44143577458863 | 0.00523560209424084 | GPR34//PTAFR// |
| GO:0005041 | low-density lipoprotein particle receptor activity | Molecular function | 2 | 14 | 382 | 17548 | 6.56245325355273 | 0.0361879703165352 | 0.78092560940837 | 1.44143577458863 | 0.00523560209424084 | VLDLR//LRP8// |
| GO:0045028 | G-protein coupled purinergic nucleotide receptor activity | Molecular function | 2 | 14 | 382 | 17548 | 6.56245325355273 | 0.0361879703165352 | 0.78092560940837 | 1.44143577458863 | 0.00523560209424084 | GPR34//PTAFR// |
| GO:0015075 | ion transmembrane transporter activity | Molecular function | 28 | 900 | 382 | 17548 | 1.42915648632926 | 0.0369807713808657 | 0.78092560940837 | 1.43202403414603 | 0.0732984293193717 | P2RX2//GRIA3//GRIN2B//ANO8//ANO3//BEST1//GABRR1//HTR3E//KCNIP3//SCN3B//SCN1A//CACNG1//LRRC38//KCNA6//KCNQ4//KCNIP2//SLC26A1//ABCC8//SLC1A6//SLC5A3//SLC39A2//SLC30A6//ATP1B4//ATP6V1G2//CTNS//SLC23A2//SLC27A4//SLC9B1// |
| GO:0022843 | voltage-gated cation channel activity | Molecular function | 7 | 143 | 382 | 17548 | 2.24867279317541 | 0.0371031950711455 | 0.78092560940837 | 1.43058869033049 | 0.0183246073298429 | GRIN2B//CACNG1//LRRC38//KCNA6//KCNQ4//KCNIP2//ABCC8// |
| GO:0001883 | purine nucleoside binding | Molecular function | 14 | 377 | 382 | 17548 | 1.70588970516755 | 0.0372604633874823 | 0.78092560940837 | 1.42875174832933 | 0.0366492146596859 | GBP4//ARFIP2//RND1//GNAO1//GNAZ//RABL3//EHD3//RHOF//RAB2A//RIT2//RASL11B//RERGL//GNA14//SEPT7// |
| GO:0033218 | amide binding | Molecular function | 12 | 308 | 382 | 17548 | 1.78975997824165 | 0.0379489462063368 | 0.78092560940837 | 1.42080027940295 | 0.031413612565445 | PEX5//NLGN1//GRIA3//KDELR1//NUP214//PPIH//MAS1//TOMM40L//CD209//HLA-DPA1//MGST1//KCNIP2// |
| GO:0032549 | ribonucleoside binding | Molecular function | 14 | 378 | 382 | 17548 | 1.7013767694396 | 0.0379616615684624 | 0.78092560940837 | 1.42065478691329 | 0.0366492146596859 | GBP4//ARFIP2//RND1//GNAO1//GNAZ//RABL3//EHD3//RHOF//RAB2A//RIT2//RASL11B//RERGL//GNA14//SEPT7// |
| GO:0000987 | proximal promoter sequence-specific DNA binding | Molecular function | 16 | 451 | 382 | 17548 | 1.62970014278915 | 0.0388409278966211 | 0.786528789906577 | 1.41071040349192 | 0.0418848167539267 | PRDM4//DLX5//ELK1//SATB2//ZNF281//GABPA//KCNIP3//ASCL1//NDN//PAX5//PLAG1//GATAD2B//PAX8//PAXBP1//CREB3L3//BHLHE40// |
| GO:0008375 | acetylglucosaminyltransferase activity | Molecular function | 4 | 60 | 382 | 17548 | 3.06247818499127 | 0.0415462860276522 | 0.809635544665603 | 1.38146779329476 | 0.0104712041884817 | EXTL2//OGT//NDST3//XYLT2// |
| GO:0016597 | amino acid binding | Molecular function | 4 | 60 | 382 | 17548 | 3.06247818499127 | 0.0415462860276522 | 0.809635544665603 | 1.38146779329476 | 0.0104712041884817 | GRIN2B//CPS1//DPYS//DDAH1// |
| GO:0022857 | transmembrane transporter activity | Molecular function | 32 | 1068 | 382 | 17548 | 1.37639468988372 | 0.0418561585591014 | 0.809635544665603 | 1.37824063249851 | 0.0837696335078534 | P2RX2//GRIA3//GRIN2B//ANO8//ANO3//BEST1//GABRR1//HTR3E//KCNIP3//SCN3B//SCN1A//CACNG1//LRRC38//KCNA6//KCNQ4//KCNIP2//SLC26A1//ABCC8//SLC1A6//SLC5A3//SLC39A2//SLC30A6//ATP1B4//SLC35A2//TOMM40L//ATP6V1G2//SLC9B1//CTNS//SLC23A2//RALBP1//SLC27A4//TIMM23// |
| GO:0000979 | RNA polymerase II core promoter sequence-specific DNA binding | Molecular function | 3 | 36 | 382 | 17548 | 3.82809773123909 | 0.0430435538158788 | 0.810220149027696 | 1.36609187909562 | 0.00785340314136126 | HEYL//H3F3B//PAX8// |
| GO:0001882 | nucleoside binding | Molecular function | 14 | 385 | 382 | 17548 | 1.67044264635888 | 0.0431367208973079 | 0.810220149027696 | 1.36515287152672 | 0.0366492146596859 | GBP4//ARFIP2//RND1//GNAO1//GNAZ//RABL3//EHD3//RHOF//RAB2A//RIT2//RASL11B//RERGL//GNA14//SEPT7// |
| GO:0032561 | guanyl ribonucleotide binding | Molecular function | 14 | 391 | 382 | 17548 | 1.64480925536616 | 0.0479538733758393 | 0.837779504533761 | 1.31917630782984 | 0.0366492146596859 | GBP4//ARFIP2//RND1//GNAO1//GNAZ//RABL3//EHD3//RHOF//RAB2A//RIT2//RASL11B//RERGL//GNA14//SEPT7// |
| GO:0019208 | phosphatase regulator activity | Molecular function | 5 | 91 | 382 | 17548 | 2.52402048213567 | 0.0485279012908975 | 0.837779504533761 | 1.31400849041748 | 0.0130890052356021 | ARPP19//PPP1R26//ANP32E//UBXN2B//PPP4R2// |
| GO:0019001 | guanyl nucleotide binding | Molecular function | 14 | 392 | 382 | 17548 | 1.64061331338818 | 0.0487918059983092 | 0.837779504533761 | 1.31165310645047 | 0.0366492146596859 | GBP4//ARFIP2//RND1//GNAO1//GNAZ//RABL3//EHD3//RHOF//RAB2A//RIT2//RASL11B//RERGL//GNA14//SEPT7// |
| GO:0016409 | palmitoyltransferase activity | Molecular function | 3 | 38 | 382 | 17548 | 3.62661890327914 | 0.0492828971324398 | 0.837779504533761 | 1.30730376976309 | 0.00785340314136126 | YKT6//ZDHHC20//GOLGA7B// |
| GO:0015077 | monovalent inorganic cation transmembrane transporter activity | Molecular function | 14 | 393 | 382 | 17548 | 1.6364387248045 | 0.0496398900156983 | 0.837779504533761 | 1.30416918941401 | 0.0366492146596859 | SCN3B//SCN1A//LRRC38//KCNA6//KCNQ4//KCNIP2//KCNIP3//ABCC8//SLC1A6//SLC5A3//ATP1B4//ATP6V1G2//SLC9B1//SLC23A2// |
